# Supplementary material for: Metabolic reprogramming and flux to cell envelope precursors in a pentose phosphate pathway mutant increases MRSA resistance to β-lactam antibiotics
Source: bioRxiv. 2023 Mar 7:2023.03.03.530734. Preprint. [Version 1] doi: 10.1101/2023.03.03.530734 (PMC10028837; doi:10.1101/2023.03.03.530734)
Supplement: Supplement 1 [file media-1.pdf]

Table S1. Bacterial strains and plasmids used in this study

Strains/plasmids	Relevant Details
***Staphylococcus aureus* strains**	
JE2	USA300 cured of p01 & p03. Parent strain from the Nebraska Transposon Mutant Library (NTML) (57)
BH1CC	MRSA clinical isolate; SCC*mec* type II; CC8 (93)
8325-4	NCTC 8325 derivative cured of prophages (94), MSSA, CC8.
RN4220	Restriction deficient derivative of *S. aureus* 8325 (94)
RN4220 pTNT	RN4220 carrying pTNT plasmid. Cm^r. (95)
NE202 (*pgl*)	JE2 *pgl*::Erm^r (SAUSA300_1902). Erm^r. (57)
pgl::Km^r	JE2 *pgl*::Km, Km^r
*pgl*R1	JE2 Δ*pgl*, VraG Gln_{394}STOP
NE202 pLI50_*pgl* (*pgl_{comp}*)	NE202 pLI50_*pgl* Erm^r, Cm^r
NE952 (*gntP*)	JE2 *gntP*. Erm^r. (57)
NE1124 (*gntK*)	JE2 *gntK*. Erm^r. (57)
NE569 (*sucC*)	JE2 *sucC*. Erm^r. (57)
NE547 (*sucA*)	JE2 *sucA*. Erm^r. (57)
NE76 (*leuB*)	JE2 *leuB*. Erm^r. (57)
NE239 (*putA*)	JE2 *putA*. Erm^r. (57)
NE1518(*gudB*)	JE2 *gudB*. Erm^r. (57)
NE70 (*vraG*)	JE2 *vraG*. Erm^r. (57)
NE645 (*vraF*)	JE2 *vraF*. Erm^r. (57)
NE481 (*graR*)	JE2 *graR*. Erm^r. (57)
NE1868 (*mecA*)	JE2 *mecA*. Erm^r. (57)
NE626 *(sdhA)*	JE2 *sdhA*. Erm^r. (57)
NE942 (*tarS*)	JE2 *tarS*. Erm^r. (57)
NE611 (*tarM*)	JE2 *tarM*. Erm^r. (57)
JE2 *pgl*::Erm^r	JE2 transductant. *pgl*::Erm^r. This study.
JE2 *gntP*::Erm^r	JE2 transductant. *gntP*::Erm^r. This study.
JE2 *gntK*::Erm^r	JE2 transductant. *gntK*::Erm^r. This study.
pgl/gntP	Km^r, Erm^r. This study.
pgl/gntK	Km^r, Erm^r. This study.
pgl/sucC	Km^r, Erm^r. This study.
pgl/sucA	Km^r, Erm^r. This study.
pgl/leuB	Km^r, Erm^r. This study.
pgl/putA	Km^r, Erm^r. This study.
pgl/gudB	Km^r, Erm^r. This study.
pgl/vraG	Km^r, Erm^r. This study.
pgl/vraF	Km^r, Erm^r. This study.
pgl/graR	Km^r, Erm^r. This study.
pgl/thrC	Km^r, Erm^r. This study.
pgl/mecA	Km^r, Erm^r. This study.
pgl/sdhA	Km^r, Erm^r. This study.
pgl/$putA_{Spec}$	Km^r, $Spec^r$. This study.
pgl/putA/vraG	Km^r, $Spec^r$, Erm^r. This study.
pgl/tarS	Km^r, Erm^r. This study.
pgl/tarM	Km^r, Erm^r. This study.
***Escherichia coli* strains**	
TOP10	(F- *mcrA* Δ(*mrr-hsdRMS-mcrBC*) φ80*lacZ*ΔM15 Δ*lacX*74 *nupG recA1 araD139* Δ(araleu)7697 *galE15 galK16 rpsL*(StrR) *endA1* λ (Invitrogen)

TOP10 pDrive	*E.coli* TOP10 carrying pDrive_*pgl*. This study.
HST08	F- ,*endA1*, *supE44*,*thi*-1,*recA1*,*relA1*,*gyrA96*,*phoA*, Φ80d*lacZ*ΔM15, Δ (*lacZYA -argF*) U169, Δ (*mrr -hsdRMS - mcrBC*), *ΔmcrA*,λ– (Takara Bio)
HST08 pLI50_*pgl*	*E. coli* HST08 carrying pLI50_*pgl*. Amp^r. This study.
IM08B	SA08BΩPN25-*hsdS* (CC8-1) (SAUSA300_0406) of NRS384 integrated between the *essQ* and *cspB* genes (96)
IM08B pKAN	IM08B carrying pKAN. Amp^r. This study.
IM08B pSPC	IM08B carrying pSPC. Amp^r. This study.
**Plasmids**	
pDrive	*E. coli* cloning vector (Qiagen)
pDrive_*pgl*	pDrive carrying *pgl* from JE2. This study.
pLI50	*E. coli* (Amp^r)-*Staphylococcus* (Cm^r)shuttle vector (86)
pLI50_*pgl*	pLI50 carrying *pgl* from JE2. *E. coli* (Amp^r)-*Staphylococcus* (Cm^r). This study.
pTNT	pJB38 with homologous DNA to *bursa aurealis* (95)
pKAN	pTNT with *aphA-3* (95)
pSPC	pTNT with *aad9* (95)

Table S2. Oligonucleotide primers used in this study.

Target gene	Primer name	Primer sequence (5'-3')
pgl	*pgl*_Fwd	TCATCCTTAATTCACCCCAATC
	*pgl*_Rev	CAGGTGTCCATTTACCACCA
	NE202_check_F	CCTAGGGTGCCGTCTCAGCCTTGGTCTTCG
	NE202_check_R	TCTGAGTTGACGCCTAATGTTGCACGAGTG
gntP	NE952_check_F	ACATCGATCATTACAGCGTTAATGCTA
gntK	NE1124_check_F	GAAGAAACAACTTGAAATGATGAAAGTG
mecA	NE1868_check_F	GGTGAAGTAGAAATGACTGAACGTC
Erm^r	Martn_ermF	TTTATGGTACCATTTCATTTTCCTGCTTTTTC
	Martn_ermR	AAACTGATTTTTAGTAAACAGTTGACGATATTC
Kan^r	KanR_fwd	GACCTAGGGGTTTCAAAATCGGCTC
	KanR_rev	GGCCTAGGTACTAAAACAATTCATCCAGTAAA
graR	NE481_check_F	GTTGCTGGTATTGAAGATTTCGG
tarS	NE942_check_F	CGATCAAGTGAGCGTTTAGTCAG
tarM	NE611_check_R	CAGCACCATTATTAGCATTAATATTCCTTG
thrC	NE886_check_R	GAATCGCTAAAATATCAGGTGCTTC
gudB	NE1518_check_R	CACCTAGTGCAGTTGATCTGTCG
sdhC	NE626_check_F	GCACATGTAGATTTGTTCTCAGTTGTACC
leuB	NE76_check_F	CTGTCACTGAAGGTACTGATGCCCAAGC
putA	NE239_check_R	GGTACTTATCAACTAATTCGTGGCTATCG
vraG	NE70_check_F	TGGTAACGCATGATCCTGTTGCAGCAAGC
vraF	NE645_check	CAGCTGATGTTCGTTGCCTTTGTCCACCAGAC
sucC	*sucC*_F	TACTCAAATCGCCATGCAGC
	*sucC*_R	AATGACTGAAACCGTTGCCC
sucA	*sucA*_F	GGCGGTAATGGACTCGGATT
	*sucA*_R	TCTACGCTATCCCCTACGTT
**Cloning primers**		
pgl	*pgl*_F	TCATCCTTAATTCACCCCAATC
	*pgl*_R	CAGGTGTCCATTTACCACCA

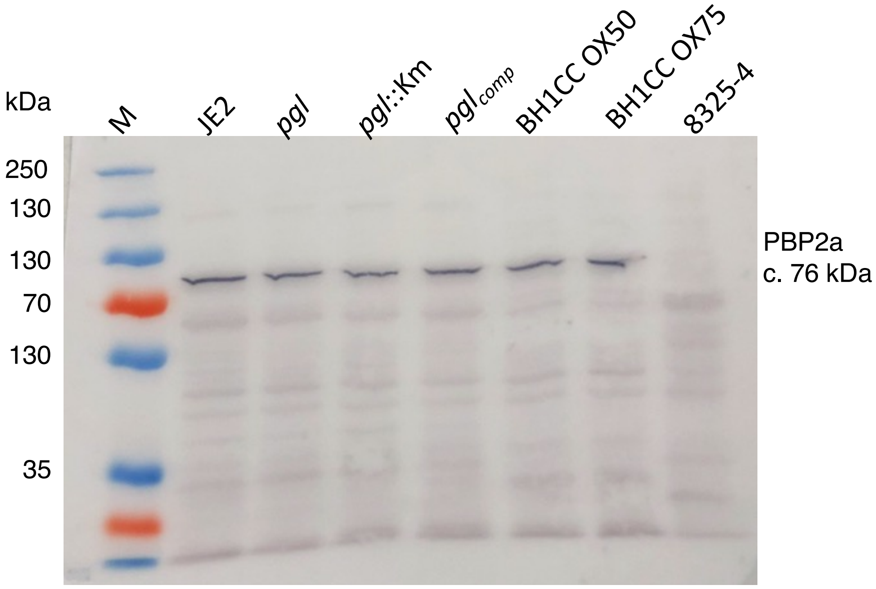

Fig. S1. Western blot of PBP2a protein in JE2, NE202 (*pgl*), *pgl*::Km^r, pgl_{comp}, HoR MRSA strain BH1CC (positive control) grown in OX 50, BH1CC grown in OX 75, MSSA strain 8325-4 (negative control). The JE2 and *pgl* strains were grown for 6 hours in TSB supplemented with 0.5 μg/ml oxacillin (OX), BHICC was grown in TSB OX 50 or 75 μg/ml, and 8325-4 which was grown in TSB with no OX. For each sample, 8 μg total protein was run on a 7.5% Tris-Glycine gel, transferred to a PVDF membrane and probed with anti-PBP2a (1:1000), followed by HRP-conjugated protein G (1:2000) and colorimetric detection with Opti-4CN Substrate kit. Three independent experiments were performed, and a representative blot is shown.

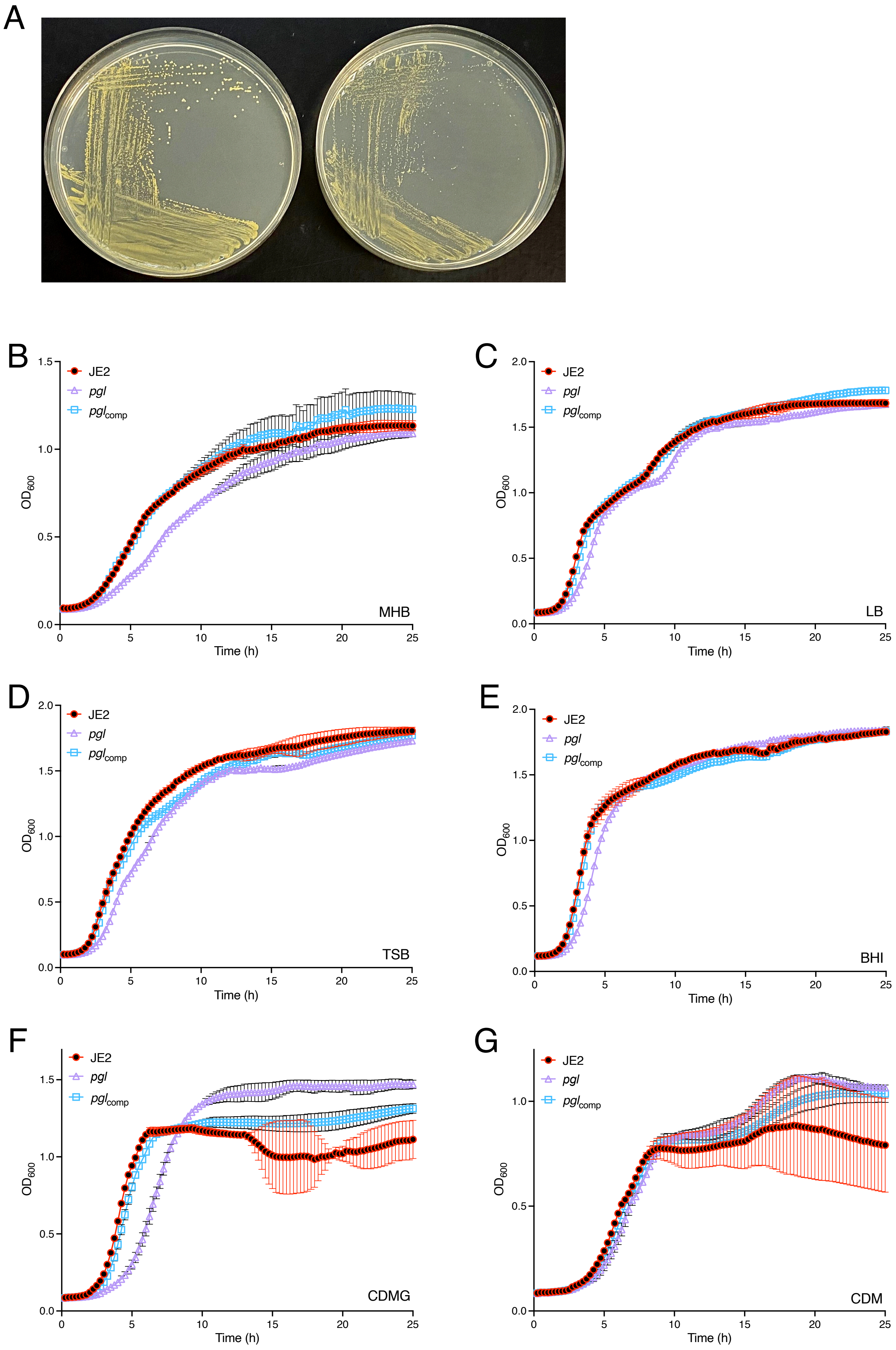

A
B
JE2
pgl
pgl comp
OD600
Time (h)
MHB
C
LB
D
TSB
E
BHI
F
CDMG
G
CDM

Fig. S2. Impact of the *pgl* mutation on growth in different culture media. A. Isolated colonies of JE2 (left) and *pgl* (right) after growth on MHA for 24 h at 37ºC. **B-G.** Growth of JE2, *pgl* and the complemented *pgl* mutant for 25 hrs at 37ºC in Mueller Hinton broth, MHB (B), Luria Bertani, LB (C), Tryptic Soya broth, TSB (D), Brain Heart Infusion, BHI (E), Chemically defined media with glucose, CDMG (F) and chemically defined media with no glucose, CDM (G). Growth (OD_{600}) was measured at 15 min intervals in a Tecan plate reader. Data are the average of 3 independent experiments plotted using GraphPad Prism V9 and error bars represent standard deviation.

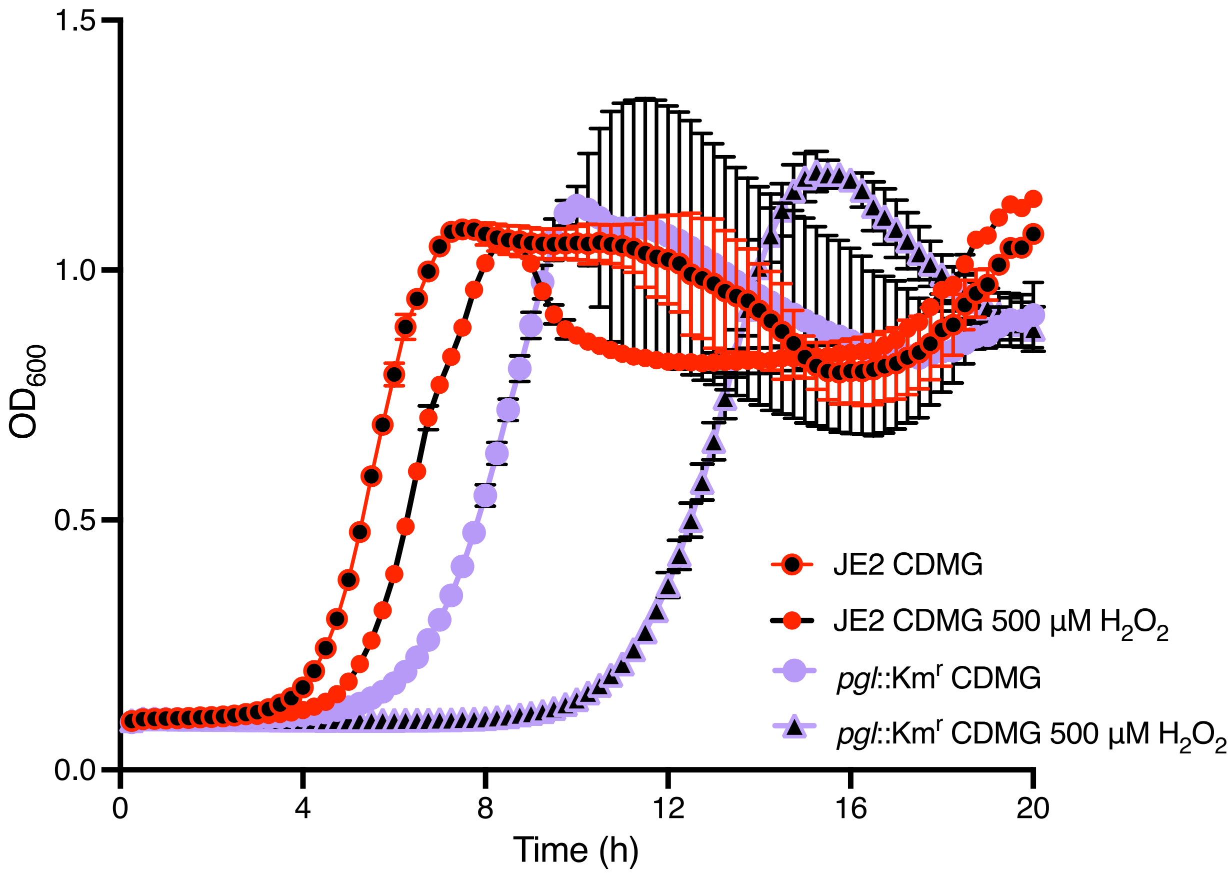

Fig. S3. Mutation of *pgl* increases sensitivity to oxidative stress. Growth of wild-type JE2 and *pgl*::Km^r for 24 hrs at 37°C in CDMG or CDMG supplemented with 500 µM H_2O_2. Growth (OD_{600}) was measured at 15 min intervals in a Tecan plate reader. Data are the average of 3 independent experiments plotted using GraphPad Prism V9 and error bars represent standard deviation.

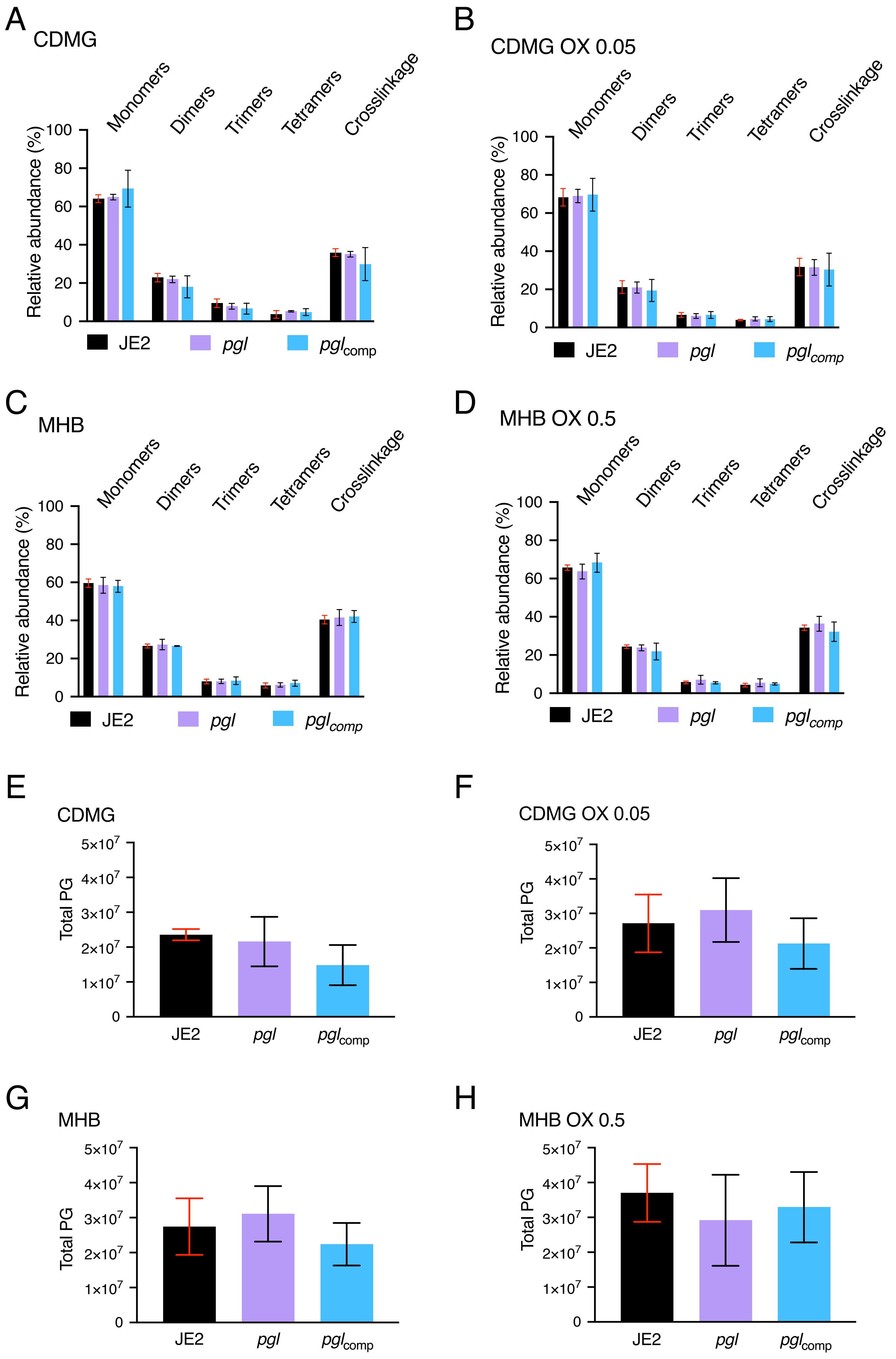
A
CDMG
Monomers
Dimers
Trimers
Tetramers
Crosslinkage
Relative abundance (%)
100
80
60
40
20
0
JE2
pgl
pgl_comp
B
CDMG OX 0.05
C
MHB
D
MHB OX 0.5
E
CDMG
Total PG
5x10^7
4x10^7
3x10^7
2x10^7
1x10^7
0
F
CDMG OX 0.05
G
MHB
H
MHB OX 0.5

Fig. S4. Comparison of peptidoglycan oligomerisation, cross-linking and total amount in JE2, *pgl* and the complemented *pgl* mutant. A-D. Relative proportions of cell wall muropeptide fractions based on oligomerization relative cross-linking efficiency in peptidoglycan extracted from JE2, *pgl* and pgl_{comp} grown to exponential phase in CDMG (A), CDMG supplemented with OX 0.05 μg/ml (B), MHB (C) and MHB supplemented with OX 0.5 mg/ml (D). **E-H.** Total peptidoglycan (PG) extracted from normalised cell extracts of JE2, *pgl* and pgl_{comp} grown to exponential phase in CDMG (E), CDMG supplemented with OX 0.05 μg/ml (F), MHB (G) and MHB supplemented with OX 0.5 μg/ml (H). The total PG content was calculated as the area below the chromatogram peaks/OD_{600} and mean and standard deviation from three/four biological repeats plotted using GraphPad Prism V9.

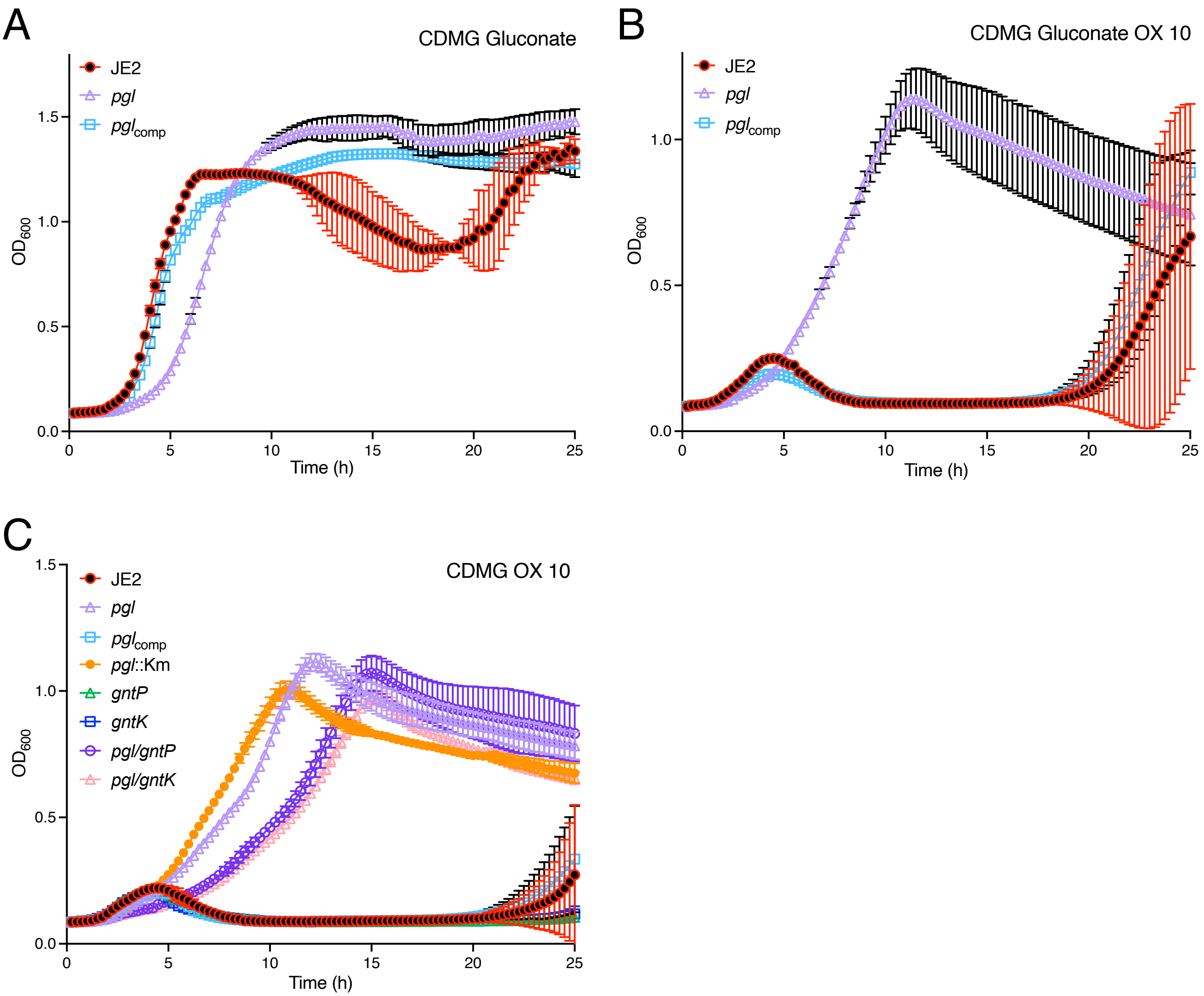

Fig. S5. Exogenous addition of D-gluconate or mutation of the gluconate shunt genes *gntP* or *gntK* did not impact growth or the increased OX resistance of the *pgl* mutant. A and B. Growth of JE2, *pgl* and the complemented *pgl* mutant for 25 hrs at 35ºC in CDMG supplemented with 5g/l potassium D-gluconate (0.5%) and no OX (A) or with both gluconate (0.5%) and OX 10 μg/ml (B). **C.** Growth of JE2, *pgl, pgl*$_{comp}$ *pgl*::Km^r, *gntP* (NE952), *gntK* (NE1124), *pgl/gntP* and *pgl/gntK* for 25 hrs at 35ºC in CDMG supplemented with OX 10 μg/ml. Growth (OD_{600}) was measured at 15 min intervals in a Tecan plate reader. Data are the average of 3 independent experiments using GraphPad Prism V9 and error bars represent standard deviation.

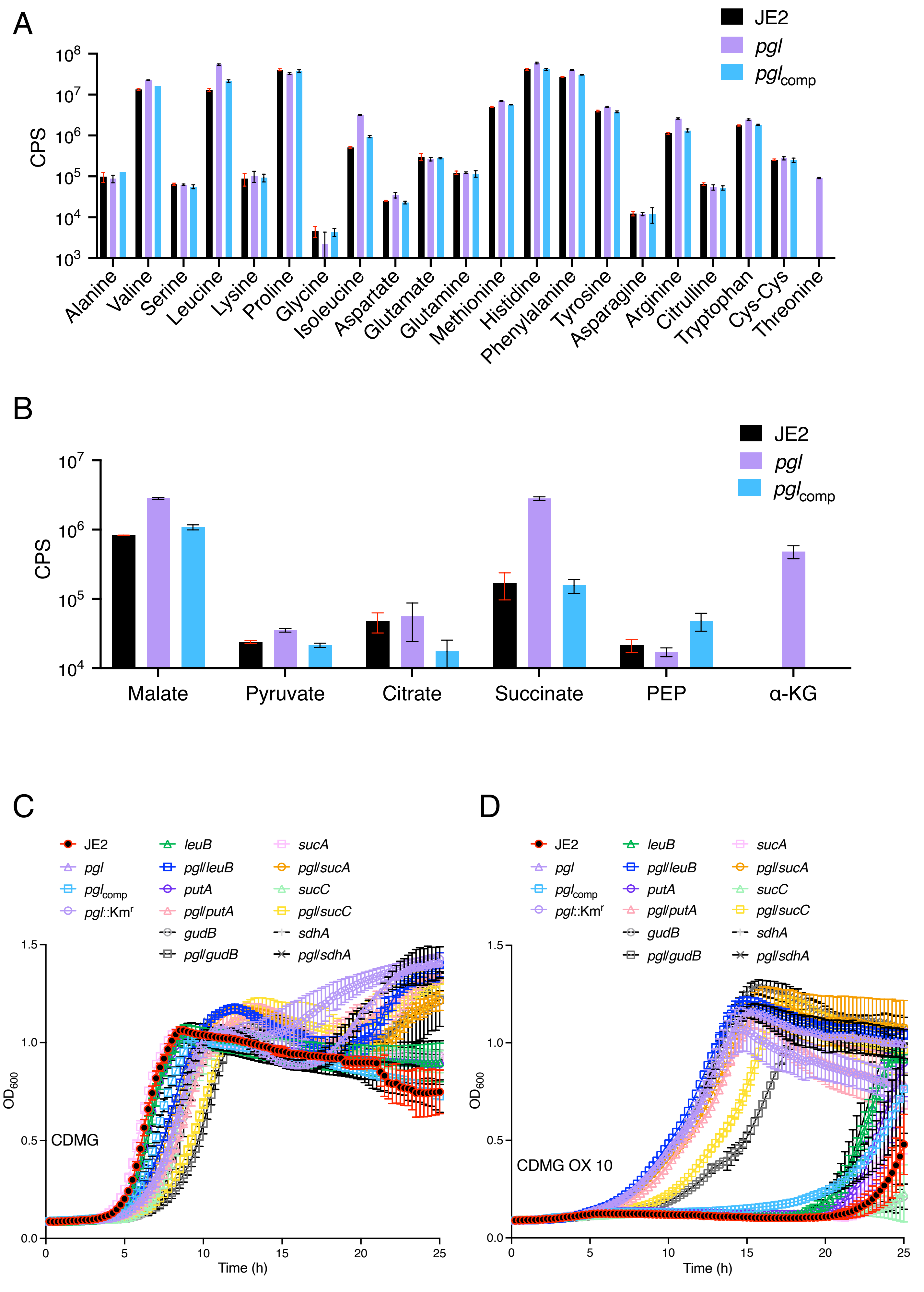

A
JE2
pgl
pglcomp
CPS
Alanine
Valine
Serine
Leucine
Lysine
Proline
Glycine
Isoleucine
Aspartate
Glutamate
Glutamine
Methionine
Histidine
Phenylalanine
Tyrosine
Asparagine
Arginine
Citrulline
Tryptophan
Cys-Cys
Threonine
B
JE2
pgl
pglcomp
CPS
Malate
Pyruvate
Citrate
Succinate
PEP
α-KG
C
JE2
pgl
pglcomp
pgl::Kmr
leuB
pgl/leuB
putA
pgl/putA
gudB
pgl/gudB
sucA
pgl/sucA
sucC
pgl/sucC
sdhA
pgl/sdhA
CDMG
OD600
Time (h)
D
CDMG OX 10
OD600
Time (h)

Fig. S6. Mutations in amino acid and TCA cycle biosynthetic genes did not reverse increased OX resistance in the *pgl* mutant. **A and B.** Comparison of amino acid (A) and TCA cycle metabolites (B) in supernatants of JE2, *pgl* and pgl_{comp} cultures grown for 7.5 h in CDMG measured by HPLC. Cell densities (OD_{600}) were normalized to each other before the cells were pelleted and supernatants collected. The data (CPS) shown are the average of three independent experiments and standard deviations are shown. **C and D.** Growth of JE2, *pgl, pgl*::Km^r, pgl_{comp}, *putA*, *gudB*, *leuB*, *sdhA*, *sucA*, *sucC*, *pgl*/*putA*, *pgl*/*gudB*, *pgl*/*leuB*, *pgl*/*sdhA*, *pgl*/*sucA* and *pgl*/*sucC* for 25 hrs at 35°C in CDMG (C) and CDMG supplemented with OX 10 μg/ml (D). Growth (OD_{600}) was measured at 15 min intervals in a Tecan plate reader. Data are the average of 3 independent experiments using GraphPad Prism V9 and error bars represent standard deviation.

A

JE2

Oxacillin (μg/ml) →

Fosfomycin (μg/ml)	0	0.25	0.5	1	2	4	8	16	32	64	128	256
128	0.053	0.05	0.051	0.048	0.049	0.046	0.048	0.046	0.049	0.05	0.053	0.056
64	0.049	0.048	0.048	0.045	0.046	0.044	0.046	0.046	0.047	0.048	0.052	0.057
32	0.054	0.046	0.046	0.046	0.047	0.045	0.046	0.044	0.044	0.046	0.049	0.055
16	0.204	0.046	0.047	0.047	0.047	0.045	0.046	0.044	0.044	0.045	0.048	0.056
8	0.347	0.048	0.047	0.048	0.05	0.047	0.045	0.044	0.043	0.044	0.047	0.055
4	0.336	0.049	0.052	0.05	0.05	0.046	0.047	0.045	0.046	0.046	0.049	0.057
2	0.368	0.056	0.054	0.055	0.061	0.05	0.051	0.046	0.046	0.046	0.05	0.056
0	0.341	0.252	0.228	0.241	0.218	0.206	0.179	0.118	0.092	0.045	0.047	0.051

B

pgl

Oxacillin (μg/ml) →

Fosfomycin (μg/ml)	0	0.25	0.5	1	2	4	8	16	32	64	128	256
128	0.07	0.053	0.049	0.048	0.049	0.047	0.05	0.047	0.049	0.051	0.057	0.059
64	0.156	0.05	0.049	0.048	0.05	0.047	0.048	0.045	0.047	0.05	0.052	0.063
32	0.241	0.05	0.052	0.051	0.051	0.05	0.054	0.053	0.05	0.049	0.05	0.064
16	0.308	0.063	0.065	0.056	0.059	0.055	0.063	0.063	0.06	0.05	0.05	0.062
8	0.32	0.091	0.094	0.097	0.092	0.089	0.1	0.095	0.086	0.049	0.05	0.069
4	0.307	0.182	0.185	0.196	0.196	0.193	0.2	0.187	0.15	0.072	0.052	0.073
2	0.319	0.273	0.261	0.261	0.27	0.269	0.257	0.24	0.21	0.126	0.054	0.061
0	0.304	0.304	0.291	0.283	0.254	0.244	0.252	0.238	0.244	0.19	0.05	0.059

Fig. S7. Mutation of *pgl* significantly increases resistance to a combination of oxacillin and fosfomycin. Checkerboard titration assays were conducted using fosfomycin and oxacillin with (A) JE2 and (B) *pgl*, grown for 24 h in Mueller Hinton 2% NaCl broth in 96-well plates. The data shown are the OD_{600} values for each well. The experiments were repeated at least three times and the data from a representative 96-well plate is shown. Green shaded boxes indicated wells in which significant growth was measured.

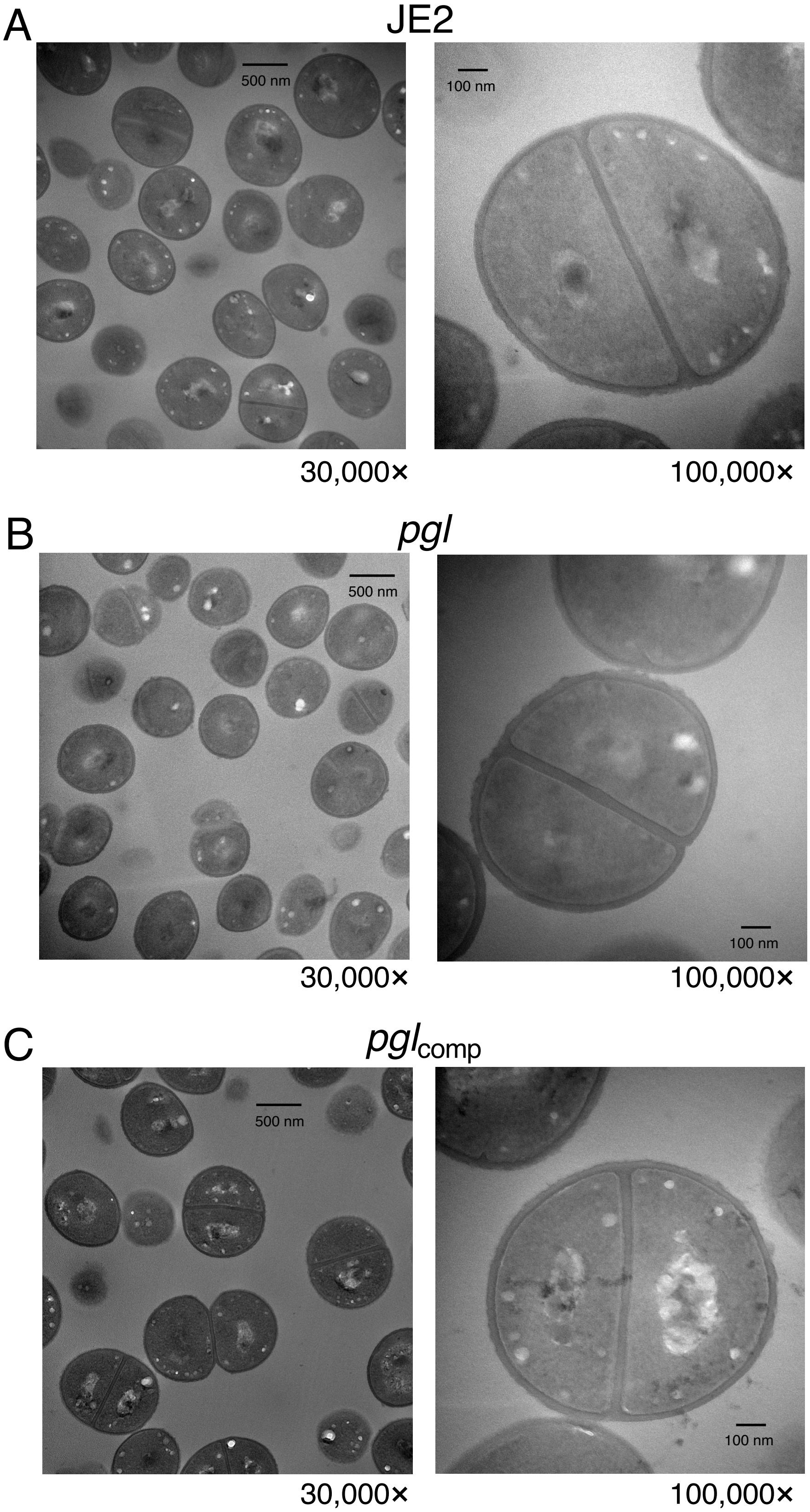

Fig. S8. Comparison of wild-type JE2 (A), *pgl* (B) and *pgl*$_{comp}$ (C) cells grown in CDMG without OX using **t**ransmission electron microscopy at 30,000× (left) and 100,000× (right) magnification. Cells were collected from exponential phase cultures grown for 4.5 h in CDMG normalized to OD_{600} = 1 in PBS before being fixed and thin sections prepared. Representative cells from each strain are shown. Scale bars represent 500 nm at 30,000× or 100 nm at 100,000× magnification.

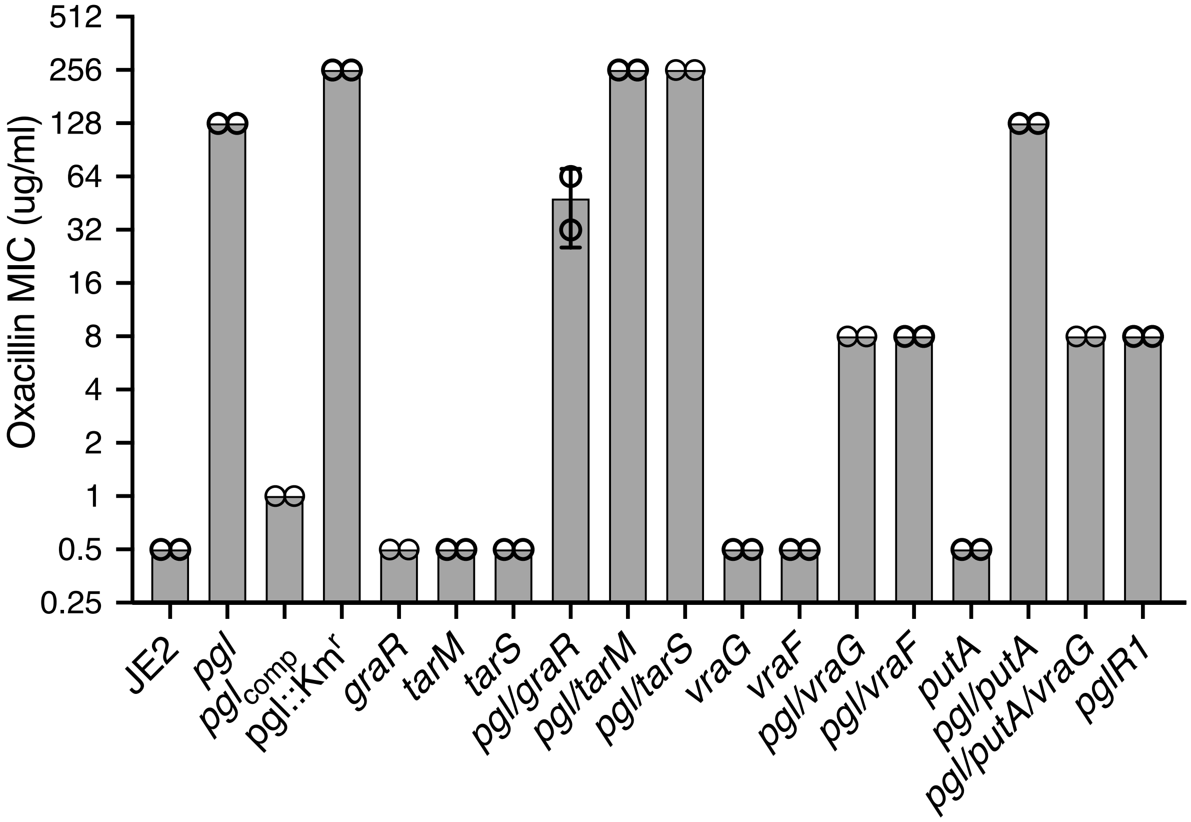

Fig. S9. Mutations in *vraF*, *vraG* and *graR* reverse the increased OX minimum inhibitory concentration (MIC) of the *pgl* mutants in CDMG. OX MICs (μg/ml) of JE2, *pgl, pgl*$_{comp}$ *pgl*::Km^r, *vraG*, *vraF*, *putA*, *graR*, *tarM*, *tarS*, *pgl*/*tarS*, *pgl*/*tarM*, *pgl*/*vraG*, *pgl*/*vraF*, *pgl*/*putA*, *pgl*/*graR*, *pgl*/*putA*/*vraG* and *pgl*R1 were measured by the broth microdilution method in CDMG. The MIC was measured in two independent experiments for each strain and variation plotted using GraphPad Prism V9 shown.
